# Comparing Behavioural Problems in Imported Street Dogs and Domestically Reared Danish Dogs—The Views of Dog Owners and Veterinarians

**DOI:** 10.3390/ani11051436

**Published:** 2021-05-17

**Authors:** Natascha Munkeboe, Amalie Lohse-Lind, Peter Sandøe, Björn Forkman, Søren Saxmose Nielsen

**Affiliations:** 1 Department of Veterinary and Animal Sciences, University of Copenhagen, DK-1870 Frederiksberg C, Denmark; tsh958@alumni.ku.dk (A.L.-L.); pes@sund.ku.dk (P.S.); bjf@sund.ku.dk (B.F.); saxmose@sund.ku.dk (S.S.N.); 2 Department of Food and Resource Economics, University of Copenhagen, DK-1958 Frederiksberg C, Denmark

**Keywords:** behaviour problems, free-ranging dog, street dog, stray dog, shelter dog, human–dog relationship

## Abstract

**Simple Summary:**

Ownerless dogs are common in parts of Southern and Eastern Europe. Some so-called street dog organisations sell them on to buyers in North European countries such as Denmark—typically via local shelters. However, with their background, the dogs may struggle to adapt to their new life as companion animals. Behavioural problems may ensue, affecting the dogs’ welfare and also presenting difficulties for the new owners. The study reported here investigated whether former street dogs imported into Denmark display more behavioural problems than dogs reared in Denmark. We examined responses to two surveys, one of Danish dog owners and one of Danish veterinarians. Our analysis appeared to confirm that street dogs display behavioural problems to a higher degree than dogs reared in Denmark. Behaviours associated with fear, stress and aggression were especially common. The extent of the behavioural problems reported by the veterinarians was greater than that reported by the dog owners, most of whom reported low levels of problems. This may be due, at least partly, to stress reactions in dogs handled by veterinarians.

**Abstract:**

Street dogs are common in southern and eastern parts of Europe. They are often adopted by people living in North European countries, including Denmark. However, these dogs may experience difficulties adjusting to their new life as companion animals, and this may in turn lead to behavioural problems and complications for owners. The objective of this study was therefore to investigate whether former street (FS) dogs display a higher degree of behavioural problems than dogs reared in Denmark (RD). Two questionnaires were developed. One was distributed to Danish dog owners and resulted in 3020 useful answers. FS dogs were found to display 9 of the 45 listed behaviours more often than RD dogs. All of these behaviours were related to fear, aggression and stress. The second questionnaire was distributed to Danish veterinarians working in small animal practices and resulted in 173 useful answers. The most commonly reported behavioural problems were fear of humans, stress and problems when the dog was left at home alone. The extent of the behavioural problems reported by the veterinarians was much greater than that reported by the dog owners which, at least partly, may be due to fear-induced reactions of the dogs when handled at the veterinary clinic.

## 1. Introduction

In Europe, street dogs are mostly found in Southern and Eastern countries [1]. The dogs are sometimes viewed as a threat to both humans and other dogs, either of which they may attack, or bite, posing a risk of disease transmission [1]. The dogs may also experience welfare problems, and to prevent the dogs from being euthanised or mistreated in the countries they live in, organisations have been created to manage these outcomes by various means, one of which is to capture and sell the dogs to owners in Northern Europe—typically via local shelters. For example, in Denmark, a total of 29 active street dog organisations have been identified (as of 2019) working in Europe, with the purpose of importing street dogs for adoption into Danish households [1]. The organisations import dogs primarily from Greece and Bosnia-Herzegovina, and, to a lesser extent, from Bulgaria, Poland, Portugal, Romania, Serbia, Spain and Turkey. Using information from nine such organisations, it has been estimated that ≥5000 former street (FS) dogs had been imported into Denmark since 2001 by these organisations alone. Data from a questionnaire completed by Danish veterinarians supported this estimate, as the 173 participating veterinarians reported handling more than 4500 FS dogs in the period from June 2018 to June 2019 [1]. In the United Kingdom, an increase in imports of FS dogs has also been reported over recent years [2,3]. From Romania alone, the number of imported FS dogs into the UK increased from 3616 dogs in 2014 to 19,487 dogs in 2019 [4]. In Norway, imports of FS dogs were banned as of 1 July 2018, meaning that these dogs were no longer considered under the rules for commercial import. To import such a dog, ownership over a minimum of six months has to be documented. The regulations were implemented in response to the perception that there was a considerable risk that diseases otherwise would be transmitted, and because it was found to be impossible to acquire sufficient health guarantees for the imported dogs [5].

Displays of undesirable behaviour are not uncommon in the domestic dog population in general [6,7]. Behavioural problems can lead to the relinquishment of dogs to shelters [7], their relocation to other homes, euthanasia and sometimes dogs being abandoned on the street. Aggression is often seen as the most severe behavioural problem [7] and is associated with relinquishment or euthanasia of the dog [8]. Fear is often reported as the most common behavioural problem in dogs: in one study, 18% of the dogs struggled with fear of people, 20% exhibited separation anxiety and 52% had noise phobia [7]. A study conducted in 2014 by the Danish Veterinary Association [9] found that 11% of dog euthanasia undertaken in veterinary clinics in Denmark was performed in order to deal with the behaviour of the dog. Among these, the following specific problems were identified as causes of euthanasia: 7% aggression, 2% fear, 1% separation-related problems and 1% other.

Imported FS dogs are believed to have encountered very varying early life experiences [4]. Street dogs are viewed as pests by some in their home countries, and they are often treated as such. It has been reported that they are sometimes kicked, hit, shot at or poisoned [1]. Many street dogs which are later adopted, live for a period in shelters. Shelters can have a negative impact on the dogs’ behaviour, as it sometimes involves social isolation and excessive noise. The capture and handling of dogs are also sometimes brutal, and the dogs may be maltreated during their time in the shelter [10]. A research commentary on imported rescue dogs also raises awareness of the potential effects and consequences of stress, which the dogs might experience during long-distance transportation to their destination of adoption [4].

To date, only limited research has been published on behavioural problems in FS dogs’ post-adoption, and on the ability of these dogs to adapt to life with their adoptive families. A Turkish study from 2014 included data collected using an online questionnaire with 75 respondents who had adopted a Turkish street dog [11]. The study suggested that although most FS dogs showed fearful behaviour when first introduced to the adoptive family, they adapted to life as companion animals. A more recent study from 2020 investigated FS dogs imported to the UK [2]. The data were collected through an online questionnaire, with the responses providing information from 3080 owners of FS dogs. The owners were asked to report on their dog’s behaviour post-adoption. The behavioural problems most commonly reported were fear of strangers and fear of noise and/or objects. It was found that the prevalence of behavioural problems in the imported FS dogs was at the same level as that in dogs adopted from shelters within the UK. The study suggested that, according to owner recollections, imported FS dogs are able to adapt quite well to life as companion animals in adoptive families. Another British study from 2020 examined owner satisfaction of imported FS dogs from Romania [12]. Most dogs (63.5%) were adopted from Romanian centres or foster homes and therefore were unseen by the owner at the point of adoption. Nevertheless, 97.4% of owners were satisfied or extremely satisfied with the adopted dog. Moreover, most owners were either extremely satisfied (62.7%) or satisfied (29.3%) with the rescue process. In regard to the behaviour of the imported Romanian FS dogs, fewer respondents (41.6%) were provided with sufficient and accurate information about the dog’s behaviour at the time of adoption. To our knowledge, no previous study has compared the level of problems found in FS and that found in locally reared dogs. However, one study [4] reports the ongoing research of UK-based behaviourists on whether behavioural issues differ between FS dogs and dogs born in the UK.

Veterinarians have been found to have a very negative view on the import of former street dogs due to the risk of importing exotic diseases [1,3].

The main aim of this study was to assess the prevalence of behavioural problems in FS dogs as compared with that found in RD dogs by: (a) comparing a range of behavioural problems (fearful behaviour, aggression, resource guarding, stress, compulsive disorders, destructive behaviours, attention-seeking behaviours and house soiling) reported by owners of FS dogs and by owners of RD dogs; (b) reporting which of these behavioural problems are most commonly seen in FS dogs by veterinarians; and (c) asking whether the degree of behavioural problems in FS dogs differs when reported by dog owners and veterinarians, respectively, and discussing any found differences. We hypothesised that FS dogs may display a higher degree of behavioural problems as a result of the negative experiences they have had on the street or in shelters and following mistreatment more generally when compared with dogs reared in Denmark (RD).

## 2. Materials and Methods

The present study utilised data from two separate questionnaires: one presented to Danish dog owners and one presented to Danish veterinarians working in small animal practices.

The term ‘former street dog’ refers to dogs satisfying two criteria: they were formerly abandoned, free-ranging or living in a shelter, and they were subsequently imported to another country by an organisation or a private individual and adopted by a dog owner in that country. We have no knowledge of the time the dogs have spent abandoned or in a shelter and have chosen the term to include them all. ‘Dogs reared in Denmark’ refers to dogs with owners, bred and reared in Denmark, as well as purebred dogs imported from foreign breeders.

### 2.1. Questionnaire to Danish Dog Owners

#### 2.1.1. Data Collection

A questionnaire was developed in SurveyXact (Ramboll, Aarhus, Denmark) to study the possible differences between FS dogs imported into Denmark and RD dogs as reflected in the owners’ assessments of the behaviour of their own dogs. The questionnaire consisted of three parts: the first covered owner information, the second requested general information about the dog and its daily life, and the third collected information on the dog’s behaviour. The first part contained questions about the demographics of the owners and their experience with dogs. Owners with multiple dogs were asked to fill in the questionnaire as it applied to their youngest dog between two and eight years of age. However, if the owner had responded to own both an FS dog and an RD dog, the questionnaire was designed to require information of only the FS dog, to secure a sufficient amount of responses on FS dogs. The owners then provided general information about the dog: breed, sex, neuter status, age and weight. Then followed questions about the acquisition of the dog, how long the owner had had it, and in the case of FS dogs, the country from which the dog had originated. Then, there was a section on training classes, on whether the dog had needed help from a behavioural expert and on the amounts of daily exercise and mental stimulation the dog was given. The last part of the questionnaire covered behavioural problems, including fearful behaviour, aggression, resource guarding, stress, compulsive disorders, destructive behaviours, attention-seeking behaviours and house soiling. This part contained 45 questions. The owners were asked to assess whether their dog displayed the relevant behaviours on a five-point scale with answers ranging across ‘Never’, ‘Rarely’, ‘At times’, ‘Often’, and ‘Always’. The answer ‘Do not know’ could also be selected.

The questionnaire was piloted with a professional dog trainer, an animal science student with expertise in dogs and four dog owners with no professional background in dog training or canine behaviour. Following adjustments, it was distributed on Facebook and a Danish online dog forum (Hundegalleri.dk, accessed on 17 November 2019). The survey was open to respondents to complete from 17 November 2019 to 14 March 2020. On Facebook, it was shared by private profiles in dog-related groups, including groups for street dog organisations and for owners of FS dogs, and in other public groups, such as groups for people living in a certain town. Membership was applied for in each of the individual groups, and all posts were then approved by the administrators of the groups before sharing. No groups declined the applications for membership or the sharing of the posts. Any dog owner residing in Denmark was permitted to answer the questionnaire. People who had previously owned a dog but did not have a dog in their care at the time of responding were allowed to fill in the questionnaire, but their answers were not included in the results.

Before answering the questionnaire, dog owners were asked to consent to anonymised use of their answers in research. All answers containing personal information were stored on encrypted USB devices. Personal information was connected with the respondent’s willingness to participate in a further experimental study and included name, e-mail address and telephone number. The study was approved by the Research Ethics Committee of SCIENCE and HEALTH, University of Copenhagen, with journal no. 504-0222/20-5000.

#### 2.1.2. Data Analysis

Data from the questionnaire on the 45 behavioural questions were extracted from SurveyXact and analysed using the statistical software R (R Core Team, Vienna, Austria). For each question, the responses were cross-tabulated for dog-type (FS or RD) with the possible answers (‘Never’, ‘Rarely’, ‘At times’, ‘Often’, ‘Always’, ‘Do not know’). A Mann–Whitney U test was performed on each behavioural question to compare the behaviour of FS and RD dogs (excluding responses ‘Do not know’), and significant *p*-values were extracted. Afterwards, the Holm–Bonferroni method was used to adjust for multiple comparisons. The results were considered statistically significant when the Holm–Bonferroni corrected *p* < 0.05. Lastly, among the behavioural questions exhibiting significant difference, a multivariable logistic analysis was performed to compare the odds of the specific behaviour in FS with the odds in RD. In this analysis, we corrected for: (a) owner’s years of experience with dogs (0–2 years; 3–5 years; 6–10 years; 11–15 years; >15 years; Do not wish to respond); (b) sex of the dog (male/female); (c) neuter status of the dog (Yes/No); (d) type of residential area (House in the country side/House in residential area/Apartment complex/Do not wish to respond); (e) dog training (Yes/Yes, train alone/Yes, but not with me/No, but did previously/No/Do not wish to respond). In the models used, the specific behaviour was dichotomised to maximise the gap between the FS and RD dogs. These analyses were performed to assess the potential influence of the co-variates. Model fit was assessed using the Akaike information criterion in all models, and the most parsimonious model was chosen.

### 2.2. Questionnaire to Danish Veterinarians

#### 2.2.1. Data Collection

A questionnaire was developed in SurveyXact to examine Danish veterinarians’ experience of the behaviour of FS dogs, and their attitude to the importation of FS dogs into Denmark. The first part of the questionnaire contained information on the demographics of the respondent, including details of the practices at which the veterinarians worked. Then followed questions about the perceived prevalence of FS dogs examined by the respondent during the last year. The next part of the questionnaire contained questions about behavioural problems seen in FS dogs. Respondents were also asked to estimate the number of FS dogs they had euthanised in the past year in response to behavioural problems. Lastly, a number of statements reporting perceptions of FS dogs were presented, and the respondents were asked whether they agreed or disagreed with these.

The questionnaire was developed using information gathered from a range of sources: interviews with organisations in Denmark working with imported street dogs, further interviews with two veterinarians with special expertise with street dogs and dialogue with a professor of parasitology (the latter related to questions about infestation not reported here). The questionnaire was piloted on a veterinarian without special insight into FS dogs and then adjusted in accordance with the feedback. It was distributed to the 1089 active practitioner members of the section of Companion Animal Veterinarians of the Danish Veterinary Association via the organisation’s general office. It was distributed first by email, on 19 June 2019, and then in a reminder, on 27 June 2019.

Before completing the questionnaire, the veterinarians were asked to consent to the anonymised use of their answers for research purposes. All answers containing personal information were stored on encrypted USB devices. The personal information included the name and location of the veterinarian’s practice. This information was needed to ensure there was a representative demographic distribution of respondents. When the findings of the study have been fully reported, the personal data will be deleted. The study was approved by the Research Ethics Committee of SCIENCE and HEALTH, University of Copenhagen, with journal no. 504-0222/20-5000.

#### 2.2.2. Data Description

The responses were tabulated for each of the questions, and no further statistical analyses were performed.

## 3. Results

### 3.1. Questionnaire to Danish Dog Owners

#### 3.1.1. Population

The questionnaire was completed by 3351 dog owners and every region of the country was represented by the participants. Of the answers, 7 were removed because the owner lived outside of Denmark, 207 were excluded because the respondent did not currently own a dog, and 8 were excluded because no dog type was reported. Furthermore, 116 respondents reported dog type as “other”, essentially leaving the data unusable. This yielded 3020 answers for further analysis. However, not all participants answered all of the questions; therefore, the total numbers of answers in the analysis are variable. The dataset is included in Supplementary materials (Tables S1 and S2).

The two groups compared in the questionnaire are RD dogs (*n* = 2372, 79%) and FS dogs (*n* = 648, 21%). The distributions of dogs in each group can be seen in Table 1, together with the dogs’ sex and neuter status.

Owner information including gender, age, residence and years of experience with dogs is also presented in Table 1. Of the participants, 92% identified as women, 7% as men and <1% as other.

The majority of RD dogs had originally been acquired from small, private breeders (59%), but some owners had purchased their dogs from a larger kennel (11%) or acquired a rehomed dog from a private home (11%). Less often, purebred dogs were imported from a foreign breeder (5%) or RD dogs were bought from a Danish shelter (2%). FS dogs were imported from 19 countries. Nine of the participants did not know the origin of their dog. The five most common countries of origin were: Greece (*n* = 286), Bosnia-Herzegovina (*n* = 137), Portugal (*n* = 82), Romania (*n* = 63) and Spain (*n* = 29). The remaining 51 dogs were from other countries. Most FS dogs (75%) were acquired through Danish organisations. However, some dogs were purchased through foreign organisations (7%) or from a private person working with street dogs (4%), and a few had been found by the owners themselves on the street in the country of origin (3%). Again, a few dogs were found by their owners at a local shelter in the country of origin (5%) or were purchased from a Danish shelter or rehomed from other families (2%).

#### 3.1.2. Behavioural Findings

The distributions of responses to 21 (of the 45) questions were initially considered significant, but nine of these response-distributions were deemed non-significant following the Holm–Bonferroni correction. The 12 questions with significant differences were then assessed using multivariable logistic regression. However, it emerged that responses to one of these questions (concerning stress at the veterinary clinic) were not significant when neuter status had been corrected for. In Table 2, the distribution of responses from the remaining 11 questions is presented; and a significant difference was found between FS dogs and RD dogs for these. 

All 45 questions can be seen in Appendix A. The 11 significant questions were assessed for the influence of covariates. Three of the questions had an impact of the covariates with the following effects: (1) stress at the veterinarian was no longer associated with FS status when corrected for neutralisation status; (2) aggression towards men was also associated with training level, but the odds ratio changed from 1.44 to 1.39 only when correcting for training level, suggesting no confounding; and (3) stress at walks was associated with sterilisation status, area and training level, but the odds ratio only changed from 1.25 to 1.23 when corrected for these factors, suggesting that no confounding was to be found here either.

It can be seen from Table 2 that the RD dogs vocalised to gain their owner’s attention and followed their owners around more often than the FS dogs did. The FS dogs displayed fearful and aggressive behaviour more often than the RD dogs and were also more likely to be fearful of men, women and children, as well as unknown dogs and unknown objects. Fear of sounds was the most common fear in FS dogs, as 28% of these dogs ‘Often’ or ‘Always’ exhibited this behaviour. The FS dogs were aggressive towards men more often than the RD dogs. Nevertheless, very high shares of the dogs in both groups (79% of FS dogs and 84% of RD dogs) never showed aggression towards men. As regards stress-related behaviour, the FS dogs showed a higher degree of stress on walks and when meeting unknown people, compared with the RD dogs.

#### 3.1.3. Effect of Breed

In the comparison of the FS and RD groups of dogs, the statistical analysis included correction for a number of owner- and dog-related factors, as mentioned in Section 2.1.2, but these did not include breed. Behavioural traits reflect the interaction of genetic and environmental factors [13,14]. The genetic factors include breed [15]. Breed may have affected the results in this study; however, with the many breed combinations, it was not possible to correct for it. The group of FS dogs consisted mainly of mixed-breed dogs, whereas the RD dog group was predominantly composed of purebred dogs (1853 purebred, 561 mixed breeds). However, we did assess (data not shown) the effect of breed. Six questions were found to have responses showing a difference >10% between FS dogs and RD dogs on the option ‘Never’, and where these questions were concerned FS dogs, RD dogs and the four most common breeds were compared. The four most common breeds were Labrador Retriever (*n* = 160), Danish/Swedish Farmdog (*n* = 116), Golden Retriever (*n* = 89) and English Cocker Spaniel (*n* = 80). The results for Labrador Retrievers, Danish/Swedish Farmdogs and Golden Retrievers appeared to be similar to those for the general RD dog group. However, the two retriever breeds always had a higher number of owners reporting ‘Never’ having observed a behavioural problem. English Cocker Spaniels showed more behavioural problems than the RD dog group in general, and, in this respect, were more similar to FS dogs. To this extent, then, an effect of breed was found.

### 3.2. Questionnaire to Danish Veterinarians

#### 3.2.1. Population

In total, 173 veterinarians, representing a response rate of 16%, responded to the questionnaire. Of the respondents, 83% identified as women and 17% as men, and the residences of the respondents were evenly distributed throughout the country. The veterinarians reported that they had examined 4549 FS dogs in the period from June 2018 to June 2019, giving an average of 26 FS dogs per veterinarian. One veterinarian reported examining 200 FS dogs in one year. Another had examined 500 in a year. The number of FS dogs euthanised due to behavioural problems was reported as 193 in total, in the one-year period.

#### 3.2.2. Behavioural Issues

**Figure 1:**
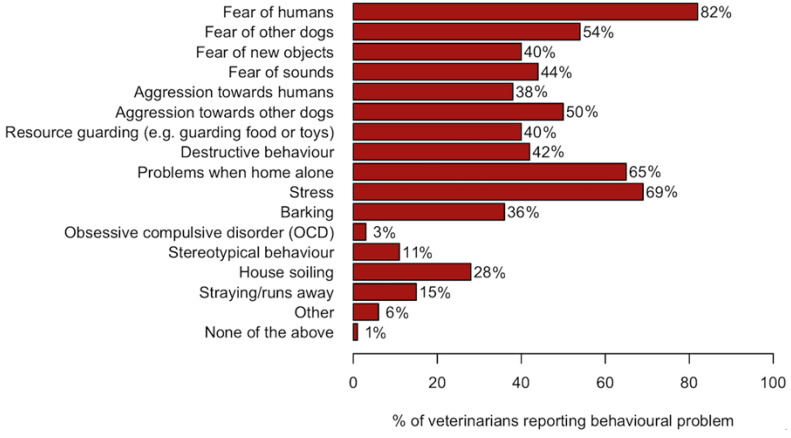
For the 15 listed behavioural problems, the percentages of veterinarians (*n* = 173) reporting that they had seen the problems in former street (FS) dogs.

The veterinarians were asked which of the 15 listed behavioural problems they often encountered in FS dogs. The options ‘Other’ and ‘None of the above’ were available in the questionnaire. The most commonly reported problems were fear of humans (82%), stress (69%) and problems when the dog was left home alone (65%) (Figure 1). Some veterinarians elaborated, when choosing the answer option ‘Other’, that some dogs were not exactly fearsome but rather nervous.

#### 3.2.3. Attitude of Veterinarians to the Importation of FS Dogs

Of the 164 veterinarians completing this part of the questionnaire, 93% stated that they agreed—Selecting ‘partially agree’, ‘agree’, or ‘strongly agree’—with the statement that FS dogs should not be imported to Denmark (Table 3). Exactly the same structure of response was obtained, though this time at 86%, for the statement that FS dogs have a higher degree of behavioural problems than RD dogs. It was also found that 80% of the veterinarians disagreed with the statement that Danish dog owners are generally well prepared to own an FS dog, with their disagreement ranging across the ‘partially disagree’, ‘disagree’, and ‘strongly disagree’ options.

## 4. Discussion

The responses to the dog-owner questionnaire appear to confirm that FS dogs display higher levels of behavioural problems related to fear, stress and aggression, compared with RD dogs. However, the differences between the two groups were not large. In contrast, the veterinarian respondents in our study seem to have encountered a much higher level of behavioural problems in FS dogs than they had in RD dogs, and they appeared to be alarmed about this.

There are various possible explanations for the contrast in the levels of behavioural problems in FS and RD dogs reported by owners and veterinarians. Dog owners may have a tendency to overlook or fail to recognise problematic behaviour in their dogs. Moreover, owners had to meet the inclusion criteria of owning a dog presently, and so the owner survey did not include owners who had surrendered their dog or had it put down due to behavioural problems or poor health. The owners represented in this survey were therefore those who had chosen to live with their dog despite any existing problems, and the existing problems were likely to be less serious than those leading to surrender or euthanasia. There is also a possibility that the owners of seriously problematic dogs simply chose not to participate in our survey.

For many dogs, a visit to the veterinarian is a stressful and fear-inducing situation [16,17]. This may partly explain the high number of veterinarians reporting fear, stress and aggression as issues they commonly encounter in FS dogs. Likewise, the high number of veterinarians reporting FS dogs’ fear of humans may be a consequence of the way the relevant dogs were handled at the clinic.

This is a reminder that, when interpreting reported behavioural problems in dogs, it is vital to bear in mind who is carrying out the reporting, and to recognise that the behaviour of the dog will change according to the setting—e.g., at home with the owner, in an outdoor situation or at the veterinary clinic.

In addition, the diseases for whose treatment some FS dogs are taken to the vet may exacerbate behavioural problems, while it is possible that the owners of well-adapted FS dogs do not seek veterinary assistance to the same degree. Newly imported dogs will, of course, very often be brought to a veterinarian for a consultation, and at this stage, the dogs may still be experiencing considerable stress. In all probability, they will not have had time to adapt to the new environment. Even though it seems that the attitude of most veterinarians is negative towards FS dogs, this might not have an effect on whether dog owners decide to adopt such a dog, as suggested in [4]. Instead, the study states the importance for veterinarians to work with clients to enhance the welfare of imported dogs, as well as trying to minimise potential risks and making epidemiological considerations.

In a separate study, based on data from the same survey as we employed, Danish veterinarians clearly had concerns about the health status of FS dogs, as 83% of them agreed (to varying degrees) that the FS dogs they saw had poorer health status than RD dogs [1]. Further, 80% of veterinarians expressed partial to strong agreement that FS dogs carry infectious diseases more often than RD dogs, and 96% stated partial to strong agreement with the statement that there is reason to be concerned about FS dogs carrying exotic diseases into Denmark [1]. This is very much in line with the findings of a survey conducted by the British Veterinary Association [3], which found that 93% of small animal veterinarians were concerned about the importation of foreign street dogs and the associated risk of exotic diseases being transmitted to animals and humans in the UK. Of the British veterinarians surveyed, 40% had experienced new, or rare, conditions associated with the import of FS dogs in the last year [3]. The uncovered health issues in FS dogs are expected to affect the general veterinarian attitude towards these dogs, along with behavioural problems.

Both the dog owners and veterinarians in our study agreed that FS dogs have higher levels of fearful behaviour, stress and aggression than RD dogs, but there were also some differences between the two survey groups as regards the most commonly reported behavioural problems. Thus, the veterinarians reported ‘Problems when the dog was left alone’ as the third most common behavioural problem in FS dogs; however, according to the dog owners, the majority of both FS and RD dogs ‘Never’ or ‘Rarely’ displayed adverse reactions (including urinating, defecating, destructive behaviour, stealing and whining) to being left at home on their own. In a Finnish study conducted on companion dogs, it was found that, when dog owners were surveyed, only 6% of the dogs displayed adverse separation-related behaviours. The study also found that noise sensitivity was the most common canine anxiety, affecting 32% of the dogs, and that fear was the second most common anxiety, affecting 29% of the dogs [18]. A study from Norway estimated noise sensitivity in 17 breeds of dogs. It found that 23% of the dogs were fearful of noises [19]. The results from both of these studies [18,19] correspond quite well with the answers reported by dog owners in our study.

Two previous studies, from the UK and Turkey, concluded that most FS dogs could adapt to life as a companion animal [2,11]. Both found that the FS dogs displayed a high frequency of fearful behaviour when first introduced to the household. One of the studies found that although many FS dogs went through a positive change post-adoption, some developed even more aggressive behaviours [11]. These differences with the findings of our study could be a sign of lower adaptability in the dogs studied, or they may reflect different expectations among the owners surveyed. In the Turkish study [11], the limited access of the dog to the house might have affected the prevalence of some behavioural problems. It is also likely that living conditions for FS dogs, both on the street and in local shelters, vary from country to country, and this—the effect of background—may impact the adaptability of these dogs. The British study [2] found that 67.5% of owners had sought assistance with their FS dog’s behavioural problems. This might indicate that most owners experienced some difficulties with the dog’s behaviour post-adoption, which is in line with the statement from Danish veterinarians, where 80% of them partially or strongly disagreed with the statement that ‘Danish dog owners are generally well prepared to own a FS dog’. Yet, 71% of British FS dog owners who had sought behavioural assistance, experienced that this had helped resolve the problems. This could indicate that a substantial share of FS dogs are trainable and can adapt to new conditions and that the FS dog owners are willing to seek assistance if needed. It is also quite promising, to say the least, that 97.4% of UK owners adopting Romanian FS dogs were satisfied or extremely satisfied with the adopted dog [12].

Compared with the above-mentioned studies, ours has the advantage of including a control group (RD dogs). This enabled us to compare the degree of behavioural problems in FS dogs with that in Danish-reared dogs. Of the two studies cited above [2,11], one examined only FS dogs from Turkey [11], and the other, from the UK [2], included street dogs that had been imported from other countries by organisations, as we also did. It should be noted that the Turkish study [11] used 22 questions and was conducted on 75 dogs. This would not have enabled the researchers to take into account multiple testing and therefore reduces the statistical power of that study. The present study and the British study [2] are more comparable in the number of questions (45 and 44, respectively). Due to the high number of questions, in the current study, we employed the Holm–Bonferroni method to correct for multiple comparisons. The British study [2] investigated responses from 3080 owners of FS dogs, as opposed to the 648 responses from FS dog owners in the present study. The British study and ours both find that a considerable number of FS dog owners report issues with fear, but they also both conclude that FS dogs are able to adapt to life as companion animals in adoptive homes. The results from the present study likewise indicate that the most prominent behavioural problems in FS dogs, as compared with RD dogs, were all related to fear. Yet, the current study disclosed a higher degree of behavioural problems in FS dogs, as compared with RD dogs, when the views of veterinarians were included. It is possible that behavioural problems in FS dogs will, in general, appear less serious when they are measured in studies based exclusively on owner reports.

We included the veterinary perspective, which is novel, compared with the previous studies [2,11] that only used the owner perspective. The inclusion of a control group in the present study was also a strength, compared with these two studies. A limitation of our study is that it is based on the subjective and retrospective reports of the dog owners and veterinarians, creating a potential for recall bias. It would have been advantageous to include independent examination of the dogs. Further, selection bias may have been unintentionally introduced due to the convenience sample of the dog owners, and there may have been a response bias towards dog owners with mainly positive or negative experiences with the adoption of an FS dog. As regards the sample of veterinarians, this is reasonably representative, since all of the respondents were members of the Danish Veterinary Association and declared that they worked with companion animals. However, the response rate among the veterinarians approached, at only 16%, was low, so it is important to remember that the results of the study may not represent the general view of veterinarians in Denmark. The veterinarians who responded to the questionnaire may have been predominantly those with a specific interest in FS dogs, developed in the course of either positive or negative experiences with these dogs, which could have elicited response bias. Furthermore, the study part including the veterinarians lacked an RD group for comparison, which is another weakness of our study.

Further research assessing the magnitude of behavioural problems and subsequent welfare issues in imported dogs would be useful. In addition, it would be a good idea to confirm the validity of the reported behavioural problems in FS dogs in countries such as Denmark, preferably by studying the dogs themselves. When interpreting behaviour in dogs, it is also important to bear in mind that displays of unwanted behaviour in dogs can be the result of health-related issues, and so further studies would benefit from the inclusion of health aspects in the questions presented to owners and veterinarians. With this addition, potential associations between behaviour and health would be picked up. Moreover, due to a found effect of breed, as covered in Section 3.1.3, further studies comparing FS dogs with other groups of dogs should aim to include this factor. Finally, our study surveyed two groups of respondents: dog owners and veterinarians. Future studies could improve our understanding of the issues presented by FS dogs by including more groups, such as behavioural experts, dog trainers and professionals working in shelters.

## 5. Conclusions

This paper presents highly conflicting evidence regarding the occurrence of behavioural problems in FS dogs adopted to Danish owners. The owners detect a level of behavioural problems involving fear, stress and aggression that is higher than that of RD dogs. However, the difference perceived by the owners is not major; FS dogs seem, according to the owners, to be able to adapt to life as companion animals in Danish homes. Contrary to this, Danish veterinarians report that they see a much higher level of behavioural problems in the imported dogs.

These conflicting reports about the level of welfare problems may, as argued above, partly be explained by the differences in the contexts in which owners and veterinarians experience with the FS dogs. However, an underlying political and ethical disagreement about whether these dogs should be allowed into Denmark also seems to be at play. Whereas the veterinarians are, to a large extent, driven by a concern about protecting Danish dogs in general against exotic diseases, the organisations behind the import and many of the prospective owners seem primarily to be motivated to save specific dogs in need. Typically, those being against the import will argue that the dogs should be helped in the countries where they live or, if that is not possible, be euthanised, whereas the other side of the debate will be more motivated by saving the lives of dogs and reject a more traditional notion of animal welfare according to which killing is not a welfare problem.

## Figures and Tables

**Table 1:** An overview of dog and owner variables distributed between the number and percentage of former street (FS) dogs and dogs reared in Denmark (RD).

Variable	Levels	FS	%FS	RD	%RD
Type	RD, purebred	0	0	1755	74
	RD, mixed breed	0	0	617	26
	FS dog	648	100	0	0
Dog sex	Male	276	43	1195	50
	Bitch	371	57	1169	49
	Missing	1	0	8	0
Neuter status	Neutered	539	83	739	32
	Intact	104	16	1621	67
	Do not know	4	1	4	0
	Missing	1	0	8	0
Owner gender	Male	44	7	180	8
	Female	604	93	2185	92
	Other	0	0	7	0
Owner age	14–38 years	181	7	828	35
	39–52 years	219	93	791	33
	53–81 years	228	0	692	29
	Missing	20	0	61	3
Residence	Countryside	223	34	748	32
	House in residential area	272	42	1100	46
	Apartment complex	150	23	512	22
	Missing	3	0	12	1
Experience with dogs	0–2 years	41	6	142	6
	3–5 years	50	8	204	9
	6–10 years	86	13	363	15
	11–15 years	83	13	367	15
	>15 years	388	60	1296	55
	Missing	0	0	0	0

**Table 2:** Distributions of responses and results from univariable Mann–Whitney U tests, with *p*-values for 11 questions with significantly different responses and the distribution of answers to questions about behaviours from owners of former street (FS) dogs and dogs reared in Denmark (RD), with six response options.

Dog Type	Behaviour	Never (%)	Rarely (%)	Sometimes (%)	Often (%)	Always (%)	Do Not Know (%)	*p*-Value
Owner home								
RD	whines for attention	29	30	30	9.9	1.5	0.3	<0.0001
FS	whines for attention	39	31	21	7.5	1.7	0.2	<0.0001
RD	follows owner	1.5	9.9	27	41	20	0.6	<0.0001
FS	follows owner	3.7	14	33	36	14	0.2	<0.0001
Fear of humans								
RD	of women	86	9.2	3.5	0.9	0.2	0.4	<0.0001
FS	of women	71	18	7	13	1.1	0.5	<0.0001
RD	of men	71	16	9.2	3.0	0.6	0.6	<0.0001
FS	of men	45	19	21	12	2.9	0.5	<0.0001
RD	of children	68	15	10	3.4	1.9	1.1	<0.0001
FS	of children	55	20	12	7.8	3.0	3.0	<0.0001
Fear of dogs, objects and sounds								
RD	unknown dogs	37	33	20	6.6	2.4	1.1	<0.0001
FS	unknown dogs	27	31	28	9.2	3.6	1.7	<0.0001
RD	unknown objects	25	44	24	5.8	1.0	0.8	<0.0001
FS	unknown objects	17	36	31	12	3.0	1.2	<0.0001
RD	of sounds	34	32	18	11	5	0.9	<0.0001
FS	of sounds	19	27	23	17	11	1.7	<0.0001
Aggression								
RD	towards men	84	8.5	3.8	1.5	0.8	1.2	0.01
FS	towards men	79	9.0	5.4	4.5	1.2	1.1	0.01
Stress								
RD	on walks	55	28	12	3.0	1.2	1.1	0.04
FS	on walks	50	27	18	4.2	0.8	0.9	0.04
RD	unknown people	56	26	11	4.7	1.2	1.0	<0.0001
FS	Unknown people	41	29	16	8.4	5.1	0.8	<0.0001

**Table 3:** The responses of 164 veterinarians asked to report their experience of behaviour in, and their general attitude to, former street (FS) dogs, on a 6-point scale.

Statement	Strongly Disagree (*n*)	Disagree (*n*)	Partially Disagree (*n*)	Partially Agree (*n*)	Agree (*n*)	Strongly Sgree (*n*)
Former street dogs have a higher degree of behavioural problems than dogs reared in Denmark.	2	8	13	25	44	72
Danish dog owners are generally well prepared to own a former street dog.	35	52	45	23	7	2
Former street dogs should not be imported to Denmark.	1	3	8	28	31	93

## Data Availability

Data are available in Supplementary materials.

## Supplementary Materials

The following are available online at https://www.mdpi.com/article/10.3390/ani11051436/s1, Table S1: Data.csv, Table S2: Variables.csv.

## Appendix A

**Table A1:** An overview of all 45 behavioural questions, including the distribution of answers for owners of former street (FS) dogs and dogs reared in Denmark (RD) between the six answer options and *p*-values.

Dog Type	Behaviour	Never (%)	Rarely (%)	Sometimes (%)	Often (%)	Always (%)	Do Not Know (%)	Total (*n*)	*p*-Value
Owner at home									
RD	urinates inside	82	9.2	2.5	0.8	5.1	0.2	2476	1
FS	urinates inside	80	11	2.6	1.4	5.1	0.3	643	1
RD	defecates inside	86	9.4	1.1	0.3	3.1	0.3	2476	1
FS	defecates inside	85	10	2.0	0.9	2.5	0.0	642	1
RD	destroys things, e.g., inventory, walls, shoes	85	12	2.3	0.4	0.4	0.6	2475	1
FS	destroys things, e.g., inventory, walls, shoes	82	13	3.9	0.5	0.6	0.2	644	1
RD	steals, e.g., food, shoes	67	19	8.4	3.1	1.3	0.4	2475	0.847
FS	steals, e.g., food, shoes	64	18	12	5.3	1.1	0.2	644	0.847
RD	vocalizes to gain attention	29	30	30	9.9	1.5	0.3	2475	0
FS	vocalizes to gain attention	39	31	21	7.5	1.7	0.2	643	0
RD	vocalizes at outside noise	16	24	33	23	5.0	0.2	2475	1
FS	vocalizes at outside noise	15	22	32	26	5.3	0.0	645	1
RD	sits or lays close to owner	0.1	1.9	14.1	58	26	0.6	2474	1
FS	sits or lays close to owner	0.3	1.9	15.4	57	26	0	644	1
RD	asks for attention	2.0	10.4	44	39	4.0	0.6	2474	1
FS	asks for attention	2.6	9.9	45	37	4.8	0	645	1
RD	follows owner around	1.5	9.9	27	41	20	0.6	2474	0
FS	follows owner around	3.7	14.0	33	36	13.5	0.2	644	0
Fear of humans									
RD	of women	86	9.2	3.5	0.9	0.2	0.4	2472	0
FS	of women	71	17.8	7.0	12.5	1.1	0.5	645	0
RD	of men	71	15.5	9.2	3.0	0.6	0.6	2472	0
FS	of men	45	18.8	20.8	11.9	2.9	0.5	645	0
RD	of children	68	15.2	10.2	3.4	1.9	1.1	2471	0
FS	of children	55	20	11.8	7.8	3.0	3.0	644	0
Fear of dogs, objects and sounds									
RD	of known dogs	83	9.9	3.1	1.2	0.5	2.6	2471	0.65
FS	of known dogs	79	13	3.4	0.9	1.1	2.5	643	0.65
RD	of unknown dogs	37	33	20	6.6	2.4	1.1	2472	0
FS	of unknown dogs	27	31	28	9.2	3.6	1.7	644	0
RD	of unknown objects	25	44	24	5.8	1.0	0.8	2473	0
FS	of unknown objects	17	36	31	12	3.0	1.2	644	0
RD	of sounds	34	32	18	11	5.0	0.9	2472	0
FS	of sounds	19	27	23	17	11	1.7	644	0
Aggression									
RD	towards women	93	4.4	1.3	0.3	0.1	1.2	2472	0.51
FS	towards women	90	7.3	1.2	0.5	0.2	1.1	643	0.51
RD	towards men	84	8.5	3.8	1.5	0.8	1.2	2472	0.01
FS	towards men	79	9.0	5.4	4.5	1.2	1.1	643	0.01
RD	towards children	85	6.8	3.2	1.7	1.0	2.1	2472	1
FS	towards children	84	7.8	3.6	1.6	0.6	2.8	642	1
RD	towards dogs from same household	73	12	3.8	0.8	0.9	10	2465	1
FS	towards dogs from same household	72	13	3.6	0.8	1.6	9.1	636	1
RD	towards dogs of same sex	44	26	18	7.9	2.6	2.3	2471	1
FS	towards dogs of same sex	48	25	15	7.0	2.0	3.4	643	1
RD	towards dogs of opposite sex	62	22	9.3	3.3	1.3	2.6	2471	0.47
FS	towards dogs of opposite sex	57	24	11	3.6	1.3	4.1	639	0.47
Resource guarding									
RD	against other dogs: food	48	23	15	6.3	3.6	3.5	2466	1
FS	against other dogs: food	47	22	16	8.4	4.7	2.5	645	1
RD	against other dogs: resting places	77	12	4.7	1.9	0.9	3.3	2466	1
FS	against other dogs: resting places	75	12	6.1	3.3	2.2	2.2	640	1
RD	against other dogs: toys	76	12	5.3	1.9	1.4	3.8	2468	1
FS	against other dogs: toys	74	14	5.0	3.3	1.2	2.3	645	1
RD	against other dogs: owner or other people in the household	88	5.8	1.9	0.6	0.4	3.4	2467	1
FS	against other dogs: owner or other people in the household	88	5.2	3.0	1.2	0.3	2.2	640	1
RD	against owner: food	64	18	9.6	3.7	1.6	3.4	2468	0.24
FS	against owner: food	69	16	7.2	3.6	1.7	2.5	643	0.24
RD	against owner: resting places	81	8.7	4.2	1.6	1.1	3.3	2467	1
FS	against owner: resting places	84	7.6	4.1	1.1	0.9	2.2	641	1
RD	against owner: toys	42	21	18	8.3	5.5	6.5	2467	1
FS	against owner: toys	43	19	18	8.9	4.1	7.4	639	1
RD	against owner: other members of the household	56	15	12	5.0	4.5	6.4	2466	1
FS	against owner: other members of the household	55	14	15	5.0	5.0	6.0	638	1
Home alone									
RD	urinates inside	84	9.1	3.0	0.8	1.2	2.1	1472	1
FS	urinates inside	84	8.9	3.1	0.9	1.1	1.9	644	1
RD	defecates inside	84	10	1.8	0.7	1.3	2.1	2471	1
FS	defecates inside	86	8.4	1.7	0.8	1.1	1.7	643	1
RD	destroys things, e.g., inventory, walls, shoes	81	11	3.6	1.3	0.7	2.2	2471	0.85
FS	destroys things, e.g., inventory, walls, shoes	77	11	7.0	1.4	1.7	1.6	643	0.85
RD	steals, e.g., food, shoes	76	13	5.9	2.0	1.0	2.8	2470	0.63
FS	steals, e.g., food, shoes	72	12	9.8	3.7	0.9	1.9	642	0.63
RD	vocalizes	46	26	13	3.7	1.2	10	2456	1
FS	vocalizes	44	23	15	6.3	1.4	9.4	635	1
Stress									
RD	at home	61	29	8	1.1	0.2	0.7	2469	1
FS	at home	59	29	10	1.4	0.5	0.6	2469	1
RD	on walks	55	28	12	3.0	1.2	1.1	2471	0,04
FS	on walks	50	27	18	4.2	0.8	0.9	645	0,04
RD	when handling or grooming	34	27	23	10	4.2	1.1	2471	1
FS	when handling or grooming	35	25	22	9.8	6.5	1.1	645	1
RD	at the veterinarian	30	27	20	13	8.6	2.2	2471	0.00001
FS	at the veterinarian	24	22	22	14	13	5.9	642	0.00001
RD	when meeting unknown dogs	36	32	19	7.6	3.4	2.0	2470	0.57
FS	when meeting unknown dogs	33	29	22	9.5	4.8	2.2	644	0.57
RD	when meeting unknown people	56	26	11	4.7	1.2	1.0	2471	0
FS	when meeting unknown people	41	29	16	8.4	5.1	0.8	645	0
Compulsive behaviour									
RD	excessive licking	59	21	13	6.0	1.3	0.6	2470	1
FS	excessive licking	63	19	10	5.0	2.3	0.9	645	1
RD	chases own tail	82	13	3.6	1.2	0.8	0.5	2469	0.07
FS	chases own tail	87	8.1	2.8	0.9	0.6	0.8	645	0.07
RD	pacing	74	19	5.3	1.0	0.6	0.7	2469	1
FS	pacing	76	16	6.5	0.9	0.8	0.6	645	1
RD	chases shadows or light	92	4.4	1.3	0.6	0.4	0.9	2468	1
FS	chases shadows or light	93	3.4	1.2	0.6	0.9	0.9	645	1

## References

[1] Nielsen S.S., Lohse-Lind A., Munkeboe N., Sandøe P. (2019). Gadehunde importeret til Danmark. Dansk Veterinærtidsskrift.

[2] Norman C., Stavisky J., Westgarth C. (2020). Importing rescue dogs into the UK: Reasons, methods and welfare considerations. Vet. Rec..

[3] The British Veterinary Association Dog Rescuers risk Harming UK Dogs and Owners by Importing Dangerous Exotic Diseases into the Country, Vets. Warn 2018. https://www.bva.co.uk/news-and-blog/news-article/dog-rescuers-risk-harming-uk-dogs-and-owners-by-importing-dangerous-exotic-diseases-into-the-country-vets-warn/.

[4] Buckley L.A. (2020). Research commentary: Imported rescue dogs: Lack of research impedes evidence-based advise to ensure the welfare of individual dogs. Vet. Rec..

[5] Mattilsynet, Norwegian Food Safety Authority (2018). Tougher Requirements for the Import of Stray Cats and Dogs. https://www.mattilsynet.no/language/english/animals/travelling_with_pets/tougher_requirements_for_the_import_of_stray_cats_and_dogs.32294.

[6] Wells D.L., Hepper P.G. (2000). Prevalence of behaviour problems reported by owners of dogs purchased from an animal rescue shelter. Appl. Anim. Behav. Sci..

[7] Martínez Á.G., Pernas G.S., Casalta J.D., Rey M.L.S., Palomino L.F.D.L.C. (2011). Risk factors associated with behavioral problems in dogs. J. Vet. Behav..

[8] Fatjó J., Amat M., Mariotti V.M., de la Torre J.L.R., Manteca X. (2007). Analysis of 1,040 cases of canine aggression in referral practice in Spain. J. Vet. Behav..

[9] The Danish Veterinary Association (2015). Årsager til aflivning af hunde. Dansk Veterinærtidsskrift.

[10] Kocis T., Gaspar C., Tibru I. (2018). Social behaviour (attachment) in stray dogs from shelters. Med. Vet..

[11] Demirbas Y.S., Emre B., Kockaya M. (2014). Integration ability of urban-free ranging dogs into adoptive families’ environment. J. Vet. Behav..

[12] Baria P., Westgarth C., Buckley L. ISAE session: South/East/Southeast Asia ‘Unseen’ adoptions: UK owner satisfaction and experiences of rescue and adoption processes of imported Romanian dogs 2020, Conference Paper. Proceedings of the International Society for Applied Ethology Global Meeting.

[13] Serpell J.A. (1987). The influence of inheritance and environment on canine behaviour: Myth and fact. J. Small Anim. Pract..

[14] Tiira K., Lohi H. (2015). Early Life Experiences and Exercise Associate with Canina Anxieties. PLoS ONE.

[15] Serpell J.A., Duffy D.L. (2016). Aspects of Juvenile and Adolescent Environment Predict Aggression and Fear in 12-Months-Old Guide Dogs. Front. Vet. Sci..

[16] Edwards P.T., Hazel S.J., Browne M., Serpell J.A., McArthur M.L., Smith B.P. (2019). Investigating risk factors that predict a dog’s fear during veterinary consultations. PLoS ONE.

[17] Hekman J.P., Karas A.Z., Dreschel N.A. (2012). Salivary cortisol concentrations and behavior in a population of healthy dogs hospitalized for elective procedures. Appl. Anim. Behav. Sci..

[18] Salonen M., Sulkama S., Mikkola S., Puurunen J., Hakanen E., Tiira K., Araujo C., Lohi H. (2020). Prevalence, comorbidity, and breed differences in canine anxiety in 13,700 Finnish pet dogs. Sci. Rep..

[19] Storengen L.M., Lingaas F. (2015). Noise sensitivity in 17 dog breeds: Prevalence, breed risk and correlation with fear in other situations. Appl. Anim. Behav. Sci..

